# Expression of hypoxic markers and their prognostic significance in soft tissue sarcoma

**DOI:** 10.3892/ol.2015.2914

**Published:** 2015-01-28

**Authors:** JEUNG IL KIM, KYUNG UN CHOI, IN SOOK LEE, YOUNG JIN CHOI, WON TACK KIM, DONG HOON SHIN, KYUNGBIN KIM, JEONG HEE LEE, JEE YEON KIM, MEE YOUNG SOL

**Affiliations:** 1 Department of Orthopedics, Pusan National University Hospital, Busan 602-739, Republic of Korea; 2 Medical Research Institute, Pusan National University Hospital, Busan 602-739, Republic of Korea; 3 Department of Pathology, Pusan National University Yangsan Hospital, Pusan National University, School of Medicine, Yangsan, Gyeongsangnam-do 626-770, Republic of Korea; 4 Department of Radiology, Pusan National University Hospital, Busan 602-739, Republic of Korea; 5 Department of Internal Medicine, Pusan National University Hospital, Busan 602-739, Republic of Korea; 6 Department of Therapeutic Radiology and Oncology, Pusan National University Hospital, Busan 602-739, Republic of Korea

**Keywords:** soft tissue sarcoma, carbonic anhydrase 9, hypoxia, hypoxia-inducible factor 1α, glucose transporter-1, vascular endothelial growth factor

## Abstract

Tumor hypoxia is significant in promoting tumor progression and resistance to therapy, and hypoxia-inducible factor 1α (HIF-1α) is essential in the adaptive response of cells to hypoxia. The aim of the present study was to investigate the expression of hypoxic markers and evaluate their prognostic significance in soft tissue sarcoma (STS). A retrospective analysis of 55 patients with STS from Pusan National University Hospital (Busan, Korea) between 1998 and 2007 was conducted, using immunohistochemistry to analyze the expression of HIF-1α, carbonic anhydrase 9 (CA9), glucose transporter-1 (GLUT1) and vascular endothelial growth factor (VEGF). The association between the overexpression of these markers and clinicopathological characteristics, including the overall survival (OS) and progression-free survival (PFS) in cases of STS, were investigated. Overexpression of HIF-1α, CA9, GLUT1 and VEGF was shown in 54.5, 32.7, 52.7 and 25.5% of tumors, respectively, and all exhibited a significant association with high French Federation of Cancer Centers (FNCLCC) grade and high American Joint Committee on Cancer (AJCC) stage. Overexpression of HIF-1α and CA9 was associated with a shorter OS and a shorter PFS. On multivariate analysis, AJCC stage and HIF-1α overexpression had independent prognostic significance. In the group receiving chemotherapy (n=27), HIF-1α overexpression was independently associated with a decreased OS. These results indicate that overexpression of HIF-1α and CA9 is associated with poor prognosis, and that HIF-1α overexpression is an independent unfavorable prognostic factor in STS.

## Introduction

In carcinogenesis, hypoxia renders a more aggressive phenotype, with increased invasiveness, proliferation and metastasis and poorer survival rate (1,2). Additionally, several clinical studies have demonstrated that hypoxia is associated with poor response to radiation and chemotherapy (3–5). Cellular adaptation to hypoxia represents a crucial step in tumor progression.

Hypoxia-inducible factor 1 (HIF-1) is a transcription factor that is critical in the adaptive cellular response to hypoxia (1,6). HIF-1 is composed of the subunits HIF-1α and HIF-1β, which are basic helix-loop-helix DNA binding proteins. HIF-1α has been described as an endogenous hypoxic marker (7), and its overexpression has been demonstrated to correlate with a poorer survival rate in patients with cancers of the cervix (8), lung (9), colon (7,10–12), endometrium (13) and ovary (14).

HIF-1α is able to induce the expression of more than 30 genes that are involved in cellular metabolism, angiogenesis, proliferation and survival. These gene products include carbonic anhydrase 9 (CA9), glucose transporter-1 (GLUT1) and vascular endothelial growth factor (VEGF) (15). CA9 is a transmembrane glycoprotein important for maintaining extracellular pH by catalyzing the reversible hydration of carbonic dioxide to carbonic acid. CA9 is overexpressed in a wide spectrum of human cancers, and has been proposed to be a potential intrinsic marker of hypoxia (10–12). The membrane-bound glycoprotein GLUT1 is responsible for facilitating glucose transport (16). Several studies have demonstrated an association between GLUT1 expression and carcinogenesis, in addition to an unfavorable prognosis in various cancers (17,18). Younes *et al* (17) reported that in bladder cancer, tumors with >10% GLUT1-positive cancer cells were more likely to have higher stage than tumors with <10% GLUT1-positive cells. These results suggested that GLUT1 expression is a marker of aggressive biological potential in patients with bladder cancer (17). Furthermore, a positive association between GLUT1 and depth of invasion, lymphatic permeation, venous invasion, lymph node metastasis, hepatic metastasis, and carcinoma stage has been reported in gastric cancers (18). VEGF acts as a potent inducer of angiogenesis, and its overexpression is also associated with a higher rate of metastases and poor outcome in a variety of human cancers. Tumors expressing high levels of VEGF were significantly more prevalent in advanced stage cancer and associated with poorer survival in ovarian and endometrial carcinomas (19,20).

Soft tissue sarcomas (STS) comprise less than 1% of all malignant tumors and consist of more than 50 histopathologic subtypes (21), many with different biological behaviors. STS is locally aggressive, and recurrence and distant metastasis are often observed. A number of prognostic factors determine tumor progression and patient outcome, including tumor grade, size, location, depth, histological type, tumor stage and presence of local relapse (22). Numerous different biological prognostic factors have been studied in STS (23). Several reports have indicated that tumor hypoxia correlates with distant metastatic spread and poor prognosis in STS. These studies measured tumor oxygenation using polarographic oxygen-sensitive electrodes, and reported that higher median pO_2_ in samples was associated with an increased risk of developing metastases, and with poorer survival (1,6). Other studies have investigated hypoxic markers in several human cancers using immunohistochemical methods as an alternative approach. Using immunohistochemistry, Maseide *et al* (24) demonstrated that the hypoxic marker CA9 indicated poor prognosis in patients with high grade STS and may be a useful marker in retrospective studies of paraffin-embedded material.

The current study aimed to determine the expression of hypoxic markers, including HIF-1α, CA9, GLUT1, and VEGF, in STS using immunohistochemistry, and to analyze the impact of overexpression on the clinicopathological features of tumor aggressiveness.

## Materials and methods

### STS tissue samples

Formalin-fixed, paraffin-embedded samples were obtained from 55 patients with STS who had undergone surgical resection at Pusan National University Hospital (Busan, Korea) between 1998 and 2007. Diagnoses were confirmed by pathological analysis using the diagnostic criteria defined in the World Health Organization (WHO) classification. Among the cases, 19 liposarcomas (LPS), 16 malignant fibrous histiocytomas (MFH), seven rhabdomyosarcomas (RMA), five leiomyosarcomas (LMS), six synovial sarcomas (SS), and two malignant peripheral nerve sheath tumors (MPNST) were recorded. Each case was evaluated according to the French Federation of Cancer Centers (FNCLCC) sarcoma group grading system and the staging system of the American Joint Committee on Cancer (AJCC) (21). Clinical information was obtained from medical records. The overall survival (OS) was calculated from the date of surgery to the date of mortality or last follow-up visit. The progression-free survival (PFS) was calculated from the date of surgery to the date of tumor relapse or progression. Written informed consent from the patients and approval from the Institutional Ethics of Pusan National University Hospital were obtained prior to the use of these materials and informed consent was obtained from all patients. Samples and clinical information were anonymized prior to statistical analysis.

### Immunohistochemistry

Each slide was deparaffinized and rehydrated according to the standard procedure (14), and was subsequently treated with 0.01 mol/l sodium citrate buffer (Ventana-Bio Tek solutions, Tucson, AZ, USA) in a laboratory microwave at 120°C for 15 min. Immunohistochemical staining was performed using the avidin-biotin peroxidase complex method with diaminobenzidine as a chromogen, using the Vectastain ABC elite kit (Vector laboratories, Burlingame, CA, USA). Rabbit polyclonal antibodies for CA9 (1:1000; Abcam, Cambridge, UK; catalog no. ab15086) and GLUT1 (1:200; Neomarkers, Fremont, CA, USA; catalog no. RB9052), and mouse monoclonal antibodies for HIF-1α (1:1000; Abcam; catalog no. ab8366) and VEGF (1:50; Neomarkers; catalog no. MS350) were used as primary antibodies. Specimens of colon adenocarcinoma and renal cell carcinoma were used as positive controls for HIF-1α and CA9, respectively, due to the known strong expression of these markers. Tumor capillaries were considered to be an internal positive control for GLUT1 and VEGF.

Immunohistochemical staining was evaluated by two independent pathologists who were blinded to the specific diagnosis and prognosis for each individual case. Expression of HIF-1α was assessed by analyzing ≥1000 tumor cells from tumor fields, and the labeling index was calculated as the percentage of labeled nuclei per total number of tumor cells that were counted. The immunoreactivity of HIF-1α was graded from 0–3+ (0, no staining; 1+, 1–25%; 2+, 26–50%; 3+, 50% nuclear staining) according to the nuclear expression, and only a grade of 3+ (>50% nuclear staining) was considered to be a positive immunohistochemical result (24,25). For GLUT1 and CA9, cases were considered positive if >10% of their cells showed distinct membranous staining. For VEGF, cases were considered positive if >10% of their cells showed distinct cytoplasmic staining (8,14).

### Statistical analysis

A statistical analysis was conducted using SPSS 17.0 software (SPSS, Chicago, IL, USA). The associations between clinicopathological variables and the expression of HIF-1α, CA9, GLUT1 and VEGF were assessed using Pearson’s χ^2^ test. OS and PFS were calculated using the Kaplan-Meier log-rank test. A multivariate analysis to assess their independent prognostic values was conducted using the Cox regression method. P<0.05 was considered to indicate a statistically significant difference.

## Results

In total, data from 55 patients were collected (mean age, 57 years; range, 1–82). The clinicopathological features observed within this sample are summarized in Table I. Stage I was classified as early stage, and stages II–IV were classified as advanced stage. None of the patients had received prior chemotherapy. In total, 31 patients (56.4%) developed either local recurrence or metastasis (progression group), whereas 24 patients (43.6%) were free of progression (progression-free group). With a median follow-up time of 38 months (range, two to 187 months), the overall survival rate was 50.9%. Chemotherapy following surgical resection was received by 27 patients. Chemotherapy consisted of mensa, doxorubicin, and ifosfamide (mesna 1.2 g/m^2^/day, doxorubicin 25 mg/m^2^/day and intravenous ifosfamide 2.0 g/m^2^/day on days 1–3; repeated every 3 weeks).

**Figure 1:**
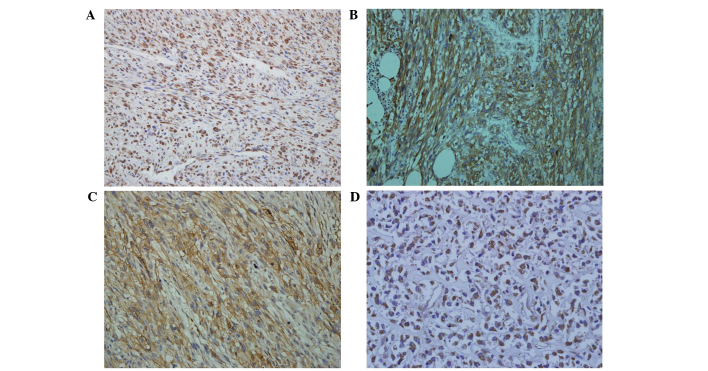
Immunohistochemical staining of HIF-1α, CA9, GLUT1 and VEGF in high grade soft tissue sarcoma. Representative cases are shown: (A) HIF-1α (magnification, ×200); (B) CA9 (magnification, ×200); (C) GLUT1 (magnificiation, ×200) and (D) VEGF (magnification, ×200). HIF-1α, hypoxia-inducible factor 1α; CA9, carbonic anhydrase 9; GLUT1, glucose transporter-1; VEGF, vascular endothelial growth factor.

Overexpression of HIF-1α, CA9, GLUT1, and VEGF was observed in 54.5% (30/55), 32.7% (18/55), 52.7% (29/55) and 25.5% (14/55) of STS samples, respectively. HIF-1α expression was recognized through the nuclear staining of positive cells, whereas CA9 and GLUT1 staining were distinct in the cell membrane. VEGF expression was observed in the cytoplasm. Representative cases of immunohistochemical staining of all markers are shown in Fig. 1.

**Figure 2:**
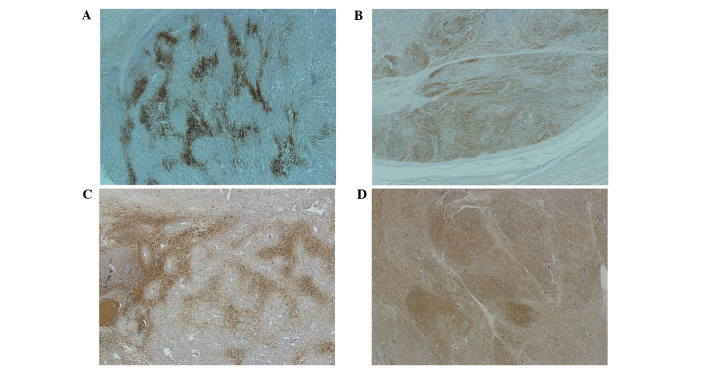
Expression pattern of CA9 and GLUT1 immunostaining: (A) Perinecrotic CA9 expression in MFH (magnification, ×40); (B) diffuse CA9 expression in LMS (magnification, ×40); (C) perinecrotic GLUT1 expression in MFH (magnification, ×40) and (D) diffuse GLUT1 expression in LMS (magnification, ×40). CA9, carbonic anhydrase 9; GLUT1, glucose transporter-1; MFH, malignant fibrous histiocytoma; LMS, leiomyosarcoma.

The correlations between clinicopathological variables and expression of HIF-1α, CA9, GLUT1 and VEGF are shown in Table II. Immunohistochemical analysis revealed a difference in the expression of HIF-1α, CA9, GLUT1 and VEGF between histological types. The rate of expression was significantly lower in LPS (HIF-1α, 36.8; CA9, 5.3; GLUT1, 15.8; and VEGF, 10.5%) compared with other types of STS, whereas MFH and LMS exhibited a higher rate of expression. The CA9 and GLUT1 positive cells were typically identified adjacent to the necrotic regions in cases of MFH with necrosis, but CA9 and GLUT1 were diffusely expressed in cases of LMS cases where no necrosis was present (Fig. 2). The expression of HIF-1α, CA9, GLUT1, and VEGF was significantly associated with a higher histological grade and advanced AJCC stage in the total cases of STS. No significant correlation between the expression of HIF-1α, CA9, GLUT1 and VEGF and tumor size or location was observed.

**Figure 3:**
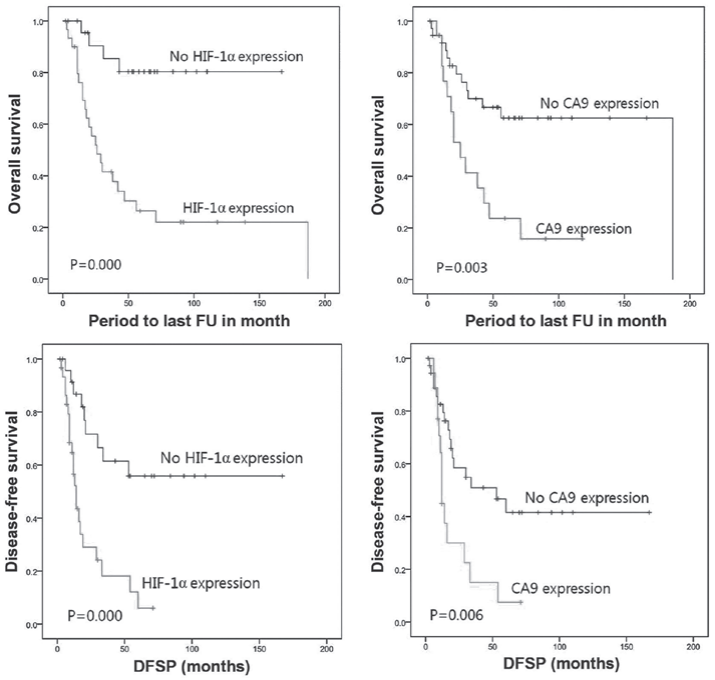
Kaplan-Meier survival curves showing disease-free survival and overall survival for soft tissue sarcoma patients with HIF-1α and CA9 expression. HIF-1α, hypoxia-inducible factor 1α; CA9, carbonic anhydrase 9; FU, follow-up; DFSP, disease-fee survival period.

Of the 30 patients exhibiting HIF-1α expression, 22 (73.3%) showed disease progression and 23 (76.7%) died from the disease, compared with only nine (36%) and four (16%), respectively, of the 25 patients who did not display HIF-1α expression. These differences were statistically significant (P=0.007 and 0.042, respectively). However, the expression of GLUT1 and VEGF was not associated with disease progression and survival (Table III). To investigate the prognostic impact of these markers in STS, Kaplan-Meier survival analyses were conducted and the differences in survival between the groups were examined. The Kaplan-Meier survival curves (Fig. 3) indicated that HIF-1α and CA9 expression had a significant impact on disease free survival (P=0.001 and 0.006) and OS (P=0.001 and 0.003). Multivariate analyses revealed that advanced AJCC stage (P=0.011) and HIF-1α expression (P=0.006) were independent prognostic markers for OS compared with early AJCC stage and no HIF-1α overexpression (Table IV). In the group receiving chemotherapy (n=27), HIF-1α expression was independently associated with shorter survival, and was an independent prognostic factor on multivariate analysis (P=0.010) (Table V).

## Discussion

Tumor hypoxia is known to affect patient prognosis as it leads to a more aggressive phenotype, with increased invasiveness, proliferation and metastasis, resulting in a poorer survival rates (1,2). HIF-1α is essential for adapting the cellular environment to hypoxia by inducing the expression of various hypoxia response molecules, including CA9, GLUT1 and VEGF. The evidence for these molecules as reliable markers of hypoxia has been reviewed elsewhere (7,10–12) and the overexpression of HIF-1α, CA9, GLUT1 and VEGF in various malignant tumors has also been demonstrated (8–14,17–20). In cervical carcinogenesis, it has been reported that HIF-1α is a marker for hypoxia-induced proliferation in the initial stage, and overexpression of GLUT1 and CA9 represent early and later events, respectively (8). Each contributes to tumor progression, greatly impacting the prognosis (11,27). Overexpression of HIF-1α and CA9 have also been shown to be powerful prognostic factors in colorectal cancers (10). Additionally, Chen *et al* (28) reported that HIF-1α affects tumor progression during breast carcinogenesis, and that GLUT1 and CA9 expression may indicate an aggressive phenotype.

Few reports have indicated that hypoxia may be a predictor of metastasis in patients with STS. Brizel *et al* (1) observed that disease-free survival was increased for patients with median tumor pO_2_ values of >10 mm Hg compared with those with median pO_2_ values of <10 mm Hg, suggesting that tumor hypoxia may be a useful marker for biologically aggressive forms of the disease. In addition, Nordsmark *et al* (6) demonstrated that patients with hypoxic tumors with a median pO_2_ of <19 mm Hg had poorer survival than those with well-oxygenated tumors, and that hypoxia was an indicator for poorer disease-specific and OS rates in patients with STS. Maseide *et al* (24) investigated the association between hypoxia and metastasis in a larger number of STS cases by conducting immunohistochemical analyses for CA9, a reliable marker of hypoxia, in paraffin-embedded tissue sections, and subsequently quantifying the CA9-positive area fraction by image analysis. The data indicated that the disease-specific and OS rates were significantly lower for patients with CA9-positive tumors than for those with CA9-negative tumors.

To the best of our knowledge, this study is the first to investigate the expression patterns of multiple hypoxic markers and their prognostic significance in STS using immunohistochemistry. Approaches to immunohistochemical evaluation for the scoring of hypoxic markers vary and may be complicated; simple and commonly used criteria were selected for use in this study in order to improve the reliability and consistency of interpretation. The overexpression of HIF-1α, CA9, GLUT1 and VEGF was observed to be significantly associated with high FNCLCC grades and high AJCC stages. The overexpression of HIF-1α and CA9 was also associated with shorter OS and shorter PFS. Furthermore, on multivariate analysis, HIF-1α overexpression exhibited independent prognostic significance.

The various HIF-1α expression patterns have different prognostic implications in certain types of cancer. In breast cancer, patients with a diffuse HIF-1α staining pattern have been demonstrated to have a significantly better prognosis than patients with perinecrotically overexpressed HIF-1α (29). Seeber *et al* (27) suggested that perinecrotic HIF-1α expression was significantly associated with a shorter disease-free survival compared with diffuse HIF-1α expression in endometrioid endometrial carcinoma. This significance of expression pattern could be explained by the fact that perinecrotic HIF-1α expression is thought to be hypoxia driven, whereas diffuse HIF-1α expression may rather be due to non-hypoxic stimuli. The results of the current study revealed a difference in the expression of HIF-1α, CA9, GLUT1 and VEGF between different histological types. The rate of expression of these molecules was significantly lower in LPS, compared with higher expression in MFH and LMS compared with other histological types. In particular, CA9 and GLUT1 expression was typically identified adjacent to the necrotic regions in cases of MFH with necrosis, but were diffusely expressed in cases of LMS where no necrosis was present. However, these expression patterns had no significant association with prognosis in STS. Further investigation is required to determine the mechanisms that result in the differing expression patterns between histological types.

Hypoxic malignant cells are more resistant to radiotherapy and chemotherapy (3–5). In advanced stage ovarian carcinoma, GLUT1 expression has been reported to be an independent prognostic factor of response to chemotherapy (30). CA9 may also be an important marker in the prediction of drug responsiveness in tongue cancer chemotherapy (31). The present study attempted to analyze the effect of HIF-1α overexpression in a group receiving chemotherapy following surgical resection, demonstrating that HIF-1α overexpression was independently associated with shorter OS in patients with STS who received chemotherapy, particularly on multivariate analysis. To the best of our knowledge, this study is the first aimed at evaluating the prognostic significance of hypoxic markers in a series of STS patients receiving chemotherapy.

Numerous studies have investigated the selective application of new treatment modalities based on targeting tumor hypoxia (15,19), reporting that hypoxic markers, including HIF-1α, CA9 and VEGF, may be specific and favorable therapeutic targets. The present study demonstrated that the expression of these molecules was common in STS. HIF-1α, CA9, GLUT1, and VEGF may therefore be useful markers to indicate aggressive phenotypes and predict prognosis, and are also potential therapeutic targets.

In conclusion, the expression of hypoxic markers, including HIF-1α, CA9, GLUT1 and VEGF is common in patients with STS and is strongly associated with tumor progression, as indicated by the significant association of their expression with higher histological grade and advanced tumor stage. In addition, the results suggest that HIF-1α overexpression is an independent unfavorable prognostic factor in STS, and may predict poor response to chemotherapy. Additional investigation of hypoxic markers, including HIF-1α, as biomarkers of aggressive tumor behavior and as novel therapeutic targets, is warranted.

## Figures and Tables

**Table I:** Clinicopathological data in 55 cases of STS.

Clinicopathological data	Cases
Age, years	
Range (median)	1–82 (57)
Gender, n	
Male	29
Female	26
Histological type, n	
LPS	19
MFH	16
RMS	7
LMS	5
SS	6
MPNST	2
Site, n	
Thigh	16
Upper arm	8
Retroperitoneum	6
Forearm	4
Lower leg	4
Head and neck	4
Back	4
Buttock	3
Others	6
Location, n	
Superficial	14
Deep	41
Tumor size, n (cm)	
<10	32
≥10	23
FNCLCC grade, n	
1	13
2	20
3	22
AJCC stage, n	
I	12
II–IV	43
Disease progression, n	
Progression-free	24
Progression	31
Overall survival, n	
Alive	28
DOD	27
Chemotherapy, n	
Yes	27
No	28
Total	55

STS, soft tissue sarcoma; LPS, liposarcoma; MFH, malignant fibrous histiocytoma; RMS, rhabdomyosarcoma; LMS, leiomyosarcoma; SS, synovial sarcoma; MPNST, malignant peripheral nerve sheath tumor; FNCLCC, French Federation of Cancer Centers; AJCC, American Joint Committee on Cancer; DOD, died of disease.

**Table II:** Association between HIF-1α, CA9, GLUT1 and VEGF expression status and clinicopathological variables (n=55).

	HIF-1α			CA9			GLUT1			VEGF		
				
Clinicopathological parameters	Positive (n=30)	Negative (n=25)	P-value	Positive (n=18)	Negative (n=37)	P-value	Positive (n=29)	Negative (n=26)	P-value	Positive (n=14)	Negative (n=41)	P-value
Histological type, n (%)			0.329			0.007			0.004			0.301
LPS	7 (36.8)	12 (63.2)		1 (5.3)	18 (94.7)		3 (15.8)	16 (84.2)		2 (10.5)	17 (89.5)	
MFH	11 (68.8)	5 (31.2)		8 (50)	8 (50)		12 (75)	4 (25)		6 (37.5)	10 (62.5)	
RMS	3 (42.9)	4 (57.1)		1 (14.3)	6 (85.7)		4 (57.1)	3 (42.9)		3 (42.9)	4 (57.1)	
LMS	4 (80)	1 (20)		4 (80)	1 (20)		4 (80)	1 (20)		2 (40)	3 (60)	
SS	4 (66.7)	2 (33.3)		3 (50)	3 (50)		4 (66.7)	2 (33.3)		1 (16.7)	5 (83.3)	
MPNST	1 (50)	1 (50)		1 (50)	1 (50)		2 (100)	0 (0)		0 (0)	2 (100)	
Tumor size, n (cm)			0.172			0.774			0.422			0.547
<10	14	17		11	20		18	13		9	22	
≥10	16	8		7	17		11	13		5	19	
Location, n			0.762			0.514			0.215			0.494
Superficial	7	7		5	9		5	9		3	11	
Deep	23	18		13	28		24	17		11	30	
Histological grade, n			0.012			0.005			0.001			0.024
Low	3	10		0	13		1	12		0	13	
High	27	15		18	24		28	14		14	28	
AJCC stage, n			0.026			0.005			0.001			0.025
I	3	9		0	12		1	11		0	12	
II to IV	27	16		18	25		28	15		14	29	

HIF-1α, hypoxia-inducible factor 1α; CA9, carbonic anhydrase 9; GLUT1, glucose transporter-1; VEGF, vascular endothelial growth factor; LPS, liposarcoma; MFH, malignant fibrous histiocytoma; RMS, rhabdomyosarcoma; LMS, leiomyosarcoma; SS, synovial sarcoma; MPNST, malignant peripheral nerve sheath tumor; AJCC, American Joint Committee on Cancer.

**Table III:** Association between HIF-1α, CA9, GLUT1 and VEGF expression status and clinical behavior of patients with soft tissue sarcoma (n=55).

	HIF-1α			CA9			GLUT1			VEGF		
				
Parameters	Positive (n=30)	Negative (n=25)	P-value	Positive (n=18)	Negative (n=37)	P-value	Positive (n=29)	Negative (n=26)	P-value	Positive (n=14)	Negative (n=41)	P-value
Disease progression, n (%)			0.007			0.042			0.422			0.067
Progression free	8 (33.3)	16 (66.7)		4 (16.7)	20 (83.3)		11 (45.8)	13 (54.2)		3 (12.5)	21 (87.5)	
Progression	22 (71)	9 (29)		14 (45.2)	17 (54.8)		18 (58.1)	13 (41.9)		11 (45.8)	20 (54.2)	
OS			0.001			0.004			0.180			0.547
Alive	7	21		4	24		12	16		6	22	
DOD	23	4		14	13		17	10		8	19	

HIF-1α, hypoxia-inducible factor 1α; CA9, carbonic anhydrase 9; GLUT1, glucose transporter-1; VEGF, vascular endothelial growth factor; OS, overall survival; DOD, died of disease.

**Table IV:** Multivariate analysis of prognostic factors in patients with soft tissue sarcomas (n=55).

Variables	Grouping	P-value	Ratio of risk	95% CI
HIF-1α	Overexpression vs. no overexpression	0.006	0.165	0.046–0.601
CA9	Positive vs. negative	0.514	0.745	0.308–1.802
Histological grade	G2–G3 vs. G1	0.208	1.822	0.716–4.636
AJCC stage	II–IV vs. I	0.011	8.096	2.480–42.707

CI, confidence interval; HIF-1α, hypoxia-inducible factor 1α; CA9, carbonic anhydrase 9; AJCC, American Joint Committee on Cancer.

**Table V:** Multivariate analysis of prognostic factors in patients with soft tissue sarcomas who received chemotherapy (n=27).

Variables	Grouping	P-value	Ratio of risk	95% CI
HIF-1α	Overexpression vs. no overexpression	0.010	0.103	0.018–0.582
CA9	Positive vs. negative	0.620	0.700	0.171–2.865
Histological grade	G2–G3 vs G1	0.507	2.381	0.184–30.849
AJCC stage	II–IV vs. I	0.442	0.539	0.111–2.607

CI, confidence interval; HIF-1α, hypoxia-inducible factor 1α; CA9, carbonic anhydrase 9; AJCC, American Joint Committee on Cancer.
